# Glutamate secretion by embryonic stem cells as an autocrine signal to promote proliferation

**DOI:** 10.1038/s41598-023-46477-2

**Published:** 2023-11-04

**Authors:** Lin Teng, Qin Qin, Ziyi Zhou, Fei Zhou, Chunyu Cao, Jian Yang, Jiawang Ding

**Affiliations:** 1 Department of Cardiology, Yichang Central People’s Hospital/The First College of Clinical Medical Sciences, China Three Gorges University, No. 183 Yiling Road, Yichang, 443003 Hubei China; 2 Institute of Cardiovascular Disease, China Three Gorges University, Yichang, 443003 Hubei China; 3 College of Basic Medical Sciences, Hubei Key Laboratory of Tumor Microencironment and Immunotherapy, China Three Gorges University, Yichang, 443000 Hubei China

**Keywords:** Cell biology, Molecular biology, Biomarkers

## Abstract

Glutamate, the major excitatory neurotransmitter in the central nervous system, has also been found to play a role in embryonic stem (ES) cells. However, the exact mechanism and function of glutamatergic signaling in ES cells remain poorly understood. In this study, we identified a glutamatergic transmission circuit in ES cells that operates through an autocrine mechanism and regulates cell proliferation. We performed biological analyses to identify the key components involved in glutamate biosynthesis, packaging for secretion, reaction, and reuptake in ES cells, including glutaminase, vesicular glutamate transporter, glutamate *N*-methyl-d-aspartate (NMDA) receptor, and cell membrane excitatory amino-acid transporter (EAAT). We directly quantified the released glutamate signal using microdialysis-high performance liquid chromatography-tandem mass spectrometry (MD–HPLC–MS–MS). Pharmacological inhibition of endogenous glutamate release and the resulting tonic activation of NMDA receptors significantly affected ES cell proliferation, suggesting that ES cells establish a glutamatergic autocrine niche via releasing and responding to the transmitter for their own regulation.

## Introduction

Glutamate is the principal excitatory neurotransmitter in the mammalian central nervous system (CNS). Glutaminase (GLS) synthesizes glutamate in glutamatergic neurons mainly from glutamine, which is then packaged into presynaptic vesicles via the vesicular glutamate transporter (VGLUT) for release into the synaptic cleft^1,2^. Glutamate then binds to its cognate receptors, including the ionotropic a-amino-3-hydroxy-5-methyl-4-isoaxazolepropionate acid (AMPA), Kainite, *N*-methyl-d-aspartate receptor (NMDA), Delta glutamate receptors, as well as the metabotropic glutamate receptor (mGluR) subtypes^3,4^. Glutamate opens the ionotropic receptor-integrated cation channel, causing an excitatory postsynaptic current (EPSC), or activates the metabotropic receptor-coupled G protein, eliciting the corresponding second messenger signal transduction^5,6^. Astrocytes take up most of the released glutamate via the cell membrane excitatory amino-acid transporter (EAAT), terminating the glutamatergic signal through sequestration of glutamate from the cleft^7^. In addition to its well-established role in neurotransmission, glutamate also exerts important functions in neurogenesis and neural development during early stages, before synapse formation. Glutamate is involved in regulating key processes such as migration, survival, differentiation, and neuritogenesis of neurons and neuronal precursors. Notably, functional glutamate receptors have been identified in neural stem/progenitor (NS/NP) cells even before the establishment of synapses, indicating the significance of glutamatergic signaling in early neural development^8–13^. Acting on its receptors, glutamate regulates the survival, proliferation, migration, and differentiation of NS/NP cells, as well as neural development^14–18^. The glutamate inputs to the NS cells in vivo could derive from afferent innervation of neurons in different brain regions^19–22^ or from the paracrine/diffuse/volume transmission^22–24^, or even from autocrine feedback of the NS cells themselves^25^. Moreover, non-neural glutamate transmission circuits were also discovered in bone osteoblasts and osteoclasts, keratinocytes, megakaryocytes, pancreatic isle cells, lung, liver, heart, kidney, adrenal tissue, and taste buds^26,27^

Functional glutamate receptors have recently been discovered to be present in ES cells^28–30^. Undifferentiated ES cells express functional mGluR, whose activation plays a role in maintaining ES cell self-renewal in culture^28^. The glutamatergic transmission system encompasses not only the receptors for signaling input but also signaling output and termination/reuptake elements. While there is ongoing investigation into the glutamate receptor-mediated signaling pathways and functions in ES cells, the origin of the signaling, i.e., the glutamate transmitter to which the receptors respond, remains unclear. Previous reports have not directly addressed the characteristics of the glutamatergic output machinery and the transmitter itself. Therefore, it is crucial and intriguing to determine whether ES cells solely express signaling input components to respond to exogenously added glutamate or if they can produce glutamate signaling, forming a glutamatergic auto-niche that regulates the cells. To examine whether ES cells possess the complete signaling machinery of glutamatergic systems and how the signaling circuit is organized, our study not only investigates the repertoire of components involved in glutamate synthesis, storage, and termination/reuptake, but also evaluates receptor function and the release of glutamate transmitter within the cells. The characterization of glutamate signaling in ES cells is essential for gaining a deeper understanding of the extrinsic regulatory mechanisms of these cells and for the development of drugs targeting glutamatergic components.

In this study, our objective was to comprehensively examine whether ES cells possess the complete signaling machinery of glutamatergic systems and elucidate the organization of the signaling circuit. We conducted a thorough investigation of the components involved in glutamate synthesis, storage, and termination/reuptake. Furthermore, combined with the assessment of glutamatergic transmission output and input components, we employed a chemical and pharmacological analysis using microdialysis-high performance liquid chromatography-tandem mass spectrometry (MD–HPLC–MS–MS) to characterize the release of glutamate into the intercellular space from ES cells. The findings from these investigations will provide valuable insights into the glutamate-mediated signaling pathway in ES cells. Moreover, they will contribute to the advancement of stem cell-based therapies and the development of drugs targeting the glutamatergic system in the future.

## Materials and methods

### Drugs and chemicals

The following reagents and chemicals were used in this study. Dulbecco’s Modified Eagle’s Medium (DMEM), knockout-Dulbecco’s modified eagle medium (KO-DMEM), fetal bovine serum (FBS), β-mercaptoethanol, l-glutamine, non-essential amino acids (NEAA), and GlutaMAX were purchased from GIBCO/Life Science (Milan, Italy). Leukemia inhibitory factor (LIF) was obtained from Chemicon (Billerica, USA). 2,3-Dioxo-6-nitro-1,2,3,4-tetrahydrobenzo[f]quinoxaline-7-sulfonamide (NBQX), d-(–)-2-Amino-5-phosphonopentanoic acid (D-AP5), and LY341495 were obtained from Tocris Bioscience (Bristol, UK). Mouse monoclonal anti-Oct-4 antibodies and mouse polyclonal anti-VGLUT2 (N-12) antibodies were purchased from Santa Cruz Biotechnology (Santa Cruz, CA, USA). Rabbit polyclonal anti-EAAT1 antibody, rabbit polyclonal anti-Gria3 antibody, rabbit polyclonal anti-Glutaminase antibody (GLS) and anti-beta-actin antibody were purchased from Abcam (Cambridge, UK). Glutamate (Glu) and all chemicals used for the preparation of the Krebs-HEPES (4-(2-hydroxyethyl)-1-piperazineethanesulfonic acid) buffer (KHB, pH 7.4) were purchased from Sigma-Aldrich (St. Louis, MO, USA). The KHB contained the following components: 135 mM NaCl, 5 mM KCl, 0.6 mM MgSO_4_, 2.5 mM CaCl_2_·2H_2_O, 1.3 mM NaH_2_PO_4_, 10 mM HEPES, 0.2 mM ascorbic acid, and 6 mM glucose. The stable isotope-labeled internal standard, Glu-d5 (4-aminobutyric-2,2,3,3,4,4-d6 acid, 99% atom D), was purchased from C/D/N Isotopes (Quebec, Canada). HPLC-grade acetonitrile (ACN), water, and formic acid were purchased from MERCK (Darmstadt, Germany). Ammonium formate was purchased from Shanghai Chemical Reagent Co., Ltd. (Shanghai, China). All other reagents were obtained from commercial sources.

### Cell lines and cell culture

The mouse ES cell line R1 was obtained from the Institute of Biochemistry and Cell Biology of the Chinese Academy of Sciences (Shanghai, China), while mouse embryonic fibroblasts (MEFs) were obtained from Sidansai Biotechnology Co., LTD (Shanghai, China). Undifferentiated ES cells were cultured on irradiated MEFs with KO-DMEM supplemented with 1000 units/mL LIF, 0.055 mM β-mercaptoethanol, 2 mM l-glutamine, 0.1 mM NEAA, and 20% FBS. ECS cells were cultured in DMEM supplemented with 10% FBS and 1% GlutaMAX. The cells were maintained at 37 °C in a humidified atmosphere containing 5% CO_2_ and 95% O_2_.

### RNA isolation and reverse transcriptase-polymerase chain reaction (RT-PCR)

Cells were plated at a density of 1 × 10^6^ cells/mL into 6 well plates and grew until they reached 70% confluence. After drug treatment, total RNA from undifferentiated ES cells was isolated with TRIzol reagent (Invitrogen, USA) according to the manufacturer’s instructions. Reverse transcription was carried out using a RevertAid First Strand cDNA Synthesis Kit (Qiagen, Germany), and the resultant single strand cDNA was stored at − 20 °C for later use in the PCR reactions. cDNA was amplified according to the following temperature profile: 94 °C for 30 s, 55 °C for 45 s, and 72 °C for 1 min. At the end of 31 cycles, the reaction was continued for an additional 10 min at 72 °C, and PCR products (15 μL) were analyzed electrophoretically on 2% agarose gels poured and run-in 1 × TAE buffer. All experiments were repeated more than three times. The primer sequences used for PCR are shown in Table 1.

**Table 1:** The primer sequences used for RT-PCR.

Gene	Primer sequences (5′–3′)	Fragment (bp)
*Grm1_F1*	aggacaataccccttcaactca	489
*Grm1_R1*	cttcacgctcatacacgaactc	
*Grm2_F1*	cctacagtgatgtctccatcca	570
*Grm2_R1*	tctggaagtaggaggcaaagtc	
*Grm3_F1*	tctgtgtggcttatcttggaga	414
*Grm3_R1*	gaggtgaagtctgtgtgtgacc	
*Grm4_F1*	gccctcaagtggaactatgtgt	455
*Grm4_R1*	cagaactcagcaaaccagatgt	
*Grm5_F1*	agccaccctcttcgttactgt	412
*Grm5_R1*	gacttctcggatgcttggatag	
*Grm6_F1*	tgactccacacgctatgacttc	521
*Grm6_R1*	agatgtttctgcggttgttctc	
*Grm7_F1*	cttggggttgggtatgtgtatt	491
*Grm7_R1*	gcgtggtggtttgtatgtagag	
*Grm8_F1*	gagaggaagaaaaccgtgaaag	578
*Grm8_R1*	acgacaaaccacacaaacactc	
*Gria1_F1*	gcttcatcactccaagttttcc	425
*Gria1_R1*	ggctccactctccttgaactta	
*Gria2_F1*	tgtatccttcatcacaccaagc	419
*Gria2_R1*	gcaggtctccatcagtaaatcc	
*Gria3_F1*	accatcagcataggtggacttt	554
*Gria3_R1*	tatcgtttttcctgccttctgt	
*Gria4_F1*	cttgtgtatgggaaagcagaga	466
*Gria4_R1*	cagccagattagcagtgtagga	
*Grid1_F1*	gaaggtatcaggaaggcaaaga	496
*Grid1_R1*	gcttgtgagcaatgtcttcatc	
*Grid2_F1*	gtggttctacgagtggtgactg	466
*Grid2_R1*	gccagagttgtgtatgggactt	
*Grik1_F1*	catctcatcctacactgccaac	460
*Grik1_R1*	cctccaccatttctctttcatc	
*Grik2_F1*	atcttcggtccttcacacagtt	564
*Grik2_R1*	gatgatggaggagacttgggta	
*Grik3_F1*	gagtctggtttgggaatactgg	445
*Grik3_R1*	ggcatagtcagggtagaggttc	
*Grik4_F1*	cattgagtatggcacgattcat	443
*Grik4_R1*	gaaccacaaagattccaccaat	
*Grik5_F1*	acatcaaggtgggtcctgag	542
*Grik5_R1*	ataggtgctggcttcacagttc	
*Grin1_F1*	cagaaacccctcagacaagttc	600
*Grin1_R1*	ggctctgctctaccactctttc	
*Grin2a_F1*	tcgggtctcatttcagtctctt	549
*Grin2a_R1*	gcagcacttcttcacattcatc	
*Grin2b_F1*	tgacttctctgtgcccttcata	581
*Grin2b_R1*	catcatctacacccctttggtt	
*Grin2c_F1*	gcactggtcttcaacaactctg	552
*Grin2c_R1*	tgtctccaacttctgtgtctcc	
*Grin2d_F1*	aatgaggatggctttctggtaa	546
*Grin2d_R1*	ctctacaaaagggacggagaag	
*Grin3a_F1*	ttgtaggggatggaaagtatgg	592
*Grin3a_R1*	gttccaaaacgaaaaccttgag	
*Grin3b_F1*	ctatccagttacacagccaacct	542
*Grin3b_R1*	aggacaaacaaccctgacaagt	
*EAAT1_F1*	gtcctgcctctcctctacttcc	478
*EAAT1_R1*	gttctcctcaatcaccgagttc	
*EAAT2_F1*	tttccagcagattcagacagtg	562
*EAAT2_R1*	gaaggtaacaggcaaagttcca	
*EAAT3_F1*	acatcaacaggacgggtaaaac	538
*EAAT3_R1*	aaatagagcaggggcagaacta	
*EAAT4_F1*	cccacatcctcagtagaaaatga	450
*EAAT4_R1*	ggaaagtgataggcagagttgc	
*EAAT5_F1*	tcctgtctgtgctctctgtcat	445
*EAAT5_R1*	gacttgataactggggtggtct	
*Gls_F1*	gtgttggtctcctcctcttgac	467
*Gls_R1*	catcatcagaatccccttgag	
*VGLUT1_F1*	ctggctatccttctgcacttct	512
*VGLUT1_R1*	ggtcccattacaaaccctgata	
*VGLUT2_F1*	gcttctggttgttggctactct	447
*VGLUT2_R1*	gaggtagcaccgtaagatttgg	
*VGLUT3_F1*	ccataccaaaggagtggctatc	481
*VGLUT3_R1*	ggtcttctggacctcacaattc	
*β-actin_F1*	gagaccttcaacaccccagc	446
*β-actin_R1*	ccacaggattccatacccaa	

### Western blot analysis

Cell lysates were prepared by sonication in RIPA buffer supplemented with a protease inhibitor cocktail (Roche, IL, USA). After centrifugation at 12,000×*g*, the supernatant was collected. Protein concentrations were determined using the BCA Protein Assay Kit (Pierce Chemical, Rockford, IL, USA). The lysates were mixed with 2.5% β-mercaptoethanol and 0.0125% bromophenol blue, followed by boiling for 5 min. Proteins were separated by 12% sodium dodecyl sulfate-polyacrylamide gel electrophoresis (SDS-PAGE) and transferred onto a polyvinylidene fluoride (PVDF) membrane (Millipore Billerica, MA, USA). The membrane was blocked with 5% (w/v) nonfat milk in Tris-buffered saline/0.05% (v/v) Tween (TBST) for 2 h and incubated overnight at 4 °C with primary antibodies at the following dilutions: anti-Oct-4 (1:500), anti-VGLUT2 (N-12) (1:500), and anti-EAAT1 (1:500), or anti-actin (1:1000) as a control. Subsequently, the membranes were incubated with corresponding secondary antibodies at room temperature for 1 h. Immunoblots were visualized and scanned using the Odyssey FC Imaging System (LI-COR Biosciences, NE, USA). All experiments were repeated more than three times.

### Immunofluorescence staining

Cells were seeded into multiple glass-bottom tissue culture plates (10 mm, Shengyou Biotechnology Co., LTD, Zhejiang, China) and cultured for 24 h. The cells were fixed with 4% formaldehyde solution for 10 min and then washed with PBS three times. The cells were then blocked with 1% BSA in PBS for 60 min at room temperature and subsequently incubated overnight at 4 °C with their corresponding primary antibodies, including anti-Oct-4 antibody (dilution 1:500), anti-GLS antibody (dilution 1:100), anti-VGLUT2 (N-12) antibody (dilution 1:100), anti-EAAT1 antibody (dilution 1:500), and anti-Gria3 antibody (dilution 1:500). In some cases, triple immunofluorescence staining was performed to investigate whether VGLUT2 and Gria3 colocalized within a single undifferentiated ES cell. After washing with PBS three times, the cells were incubated with the corresponding secondary antibody for 1 h at room temperature. The cells were then washed with PBS three times and subjected to laser confocal microscopy (Zeiss LSM 710, Deutschland, Germany) analysis.

### Measurement of free cytosolic calcium concentrations ([Ca^2+^]i)

Changes in [Ca^2+^]i were monitored using Fluo-4 AM (Dojindo, Japan) initially dissolved in dimethylsulfoxide and stored at − 20 °C. Cells were seeded into 35 mm culture dishes and rinsed twice with Krebs-Hepes buffered solution. They were then incubated in Krebs-Hepes buffered solution containing 1 μM fluo-4 AM with 5% CO_2_–95% O_2_ at 37 °C for 10 min, rinsed twice with the KHB solution, mounted on a perfusion chamber, and scanned at 1 s interval using confocal microscopy (UltraVIEW VoX, PerkinElmer, 400 ×). The fluorescence was excited at 488 nm, and the emitted light was observed at 510 nm. The imaging protocol typically begins with capturing initial fluorescence image lasting 30–60 s. Following this, we introduce the necessary drugs and proceed with image acquisition. An increase in the fluorescence ratio at the same point indicated an increase in free intracellular Ca^2+^ concentration. The analyses of [Ca^2+^]i were performed in a single cell, and the results were expressed as the fluorescent intensity (F/F0%, arbitrary unit, where F is the fluorescence captured at a particular time and F0 is the initial fluorescence image captured). We analyze the dynamics of [Ca^2+^]i using Volocity Demo 6.0 (PerkinElmer Inc). All experiments were repeated more than three times.

### Measurement of glutamate concentration by MD–HPLC–MS–MS

ES cells devoid of MEFs were plated in 10-cm diameter cell culture dishes at a density of 3 × 10^6^ cells/well. After attachment, the cells were washed three times with 10 mL KHB to remove endogenous glutamate in the old medium and then incubated in 8 mL of fresh KHB. For the time course experiments, the supernatants (120 μL) comprising glutamate released by the cells were collected at time points of 2, 3, 5, 10, 15, 30, 60, and 120 min after the addition of fresh KHB. An equal volume of fresh KHB was added back into the corresponding culture dishes after each sampling. Three or two independent experiments were conducted in duplicate for ES cells, respectively. All samples were stored at − 70 °C until analysis. All experiments were repeated more than three times. Glutamate concentrations were determined by LC–MS/MS.

### Cell proliferation assay

Cell proliferation was determined using the flow cytometry technology and Click-iT EdU Cell Proliferation Assay kit (Invitrogen, USA). Cells were seeded in 96-well plates at a density of 5000 cells/well and allowed to adhere for 24 h. The cells were then treated with NBQX, D-AP5, and LY341495 at various concentrations for 24 h. After treatment, 20 μM EdU was added to each well, and the cells were incubated for an additional 30 min at 37 °C. Cells were fixed with 3.7% paraformaldehyde for 15 min and permeabilized with 0.5% Triton X-100 for 20 min. EdU-labeled cells were visualized using Alexa Fluor 594 dye according to the manufacturer's instructions. The cells were counterstained with DAPI for 5 min at room temperature. The percentage of proliferating cells was calculated by dividing the number of EdU-positive cells by the total number of DAPI-stained cells in six randomly selected microscopic fields per well and identification and analysis of DAPI, EdU and Oct4 by high content screening. All experiments were repeated more than three times.

### Cell cycle analysis by flow cytometry

Cells (5 × 10^5^) were seeded in 6-well plates and grown until reaching 80% confluence. After exposure to NBQX, D-AP5, and LY341495, the cells were incubated for an additional 24 h. Detached cells were then washed twice and fixed with 500 μL of 70% ethanol at 4 °C for 24 h. Following a PBS wash, the cells were resuspended in PBS containing 50 μg RNAase A/mL and 2 mg/mL propidium iodide (PI) and incubated at 37 °C for 30 min. The samples were kept on ice and analyzed immediately using a FACS Calibur flow cytometer (BD, USA).

### Statistical analysis

The results were expressed as mean ± standard error of the mean (SEM) and analyzed using one-way analysis of variance (ANOVA) followed by post-hoc Tukey’s test for multiple comparisons. Comparisons between two groups were made using an unpaired two-tailed *t*-test, assuming equal variances. A p-value of less than 0.05 was considered statistically significant.

## Results

### Glutamatergic signaling components are expressed in ES cells

**Figure 1:**
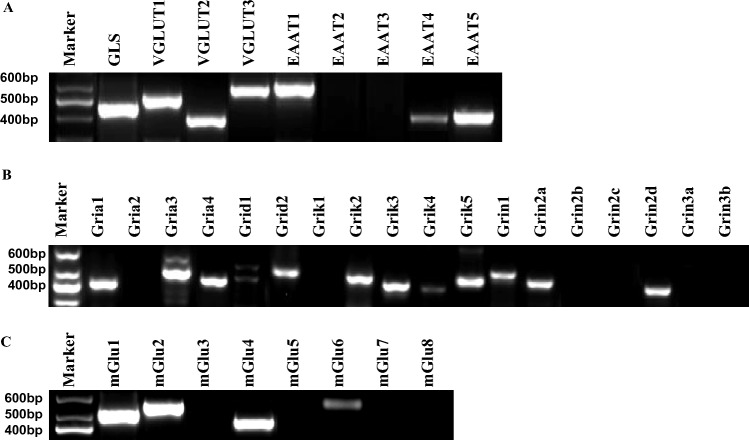
Expression of glutamatergic transmission components in ES cells. RT-PCR analysis for glutamatergic signaling output, input and reuptake components of ES cells. (**A**) Glutamatergic signaling output components GLS, VGLUT1, VGLUT2, and VGLUT3, signaling reuptake components glutamate transporters EAAT1, EAAT4, and EAAT5 were transcribed in ES cells. (**B**,**C**) Glutamatergic signaling input components, including AMPA receptor subunits Gria1/3/4, Delta receptor subunit Grid1/2, Kainate receptor subunits Grik2/3/4/5, NMDA receptor subunits Grin1/2a/2d, and mGluR subtypes Grm1/2/4/6, were transcribed in ES cells. All experiments were repeated more than three times. Original gels are presented in Supplementary Fig. 1.

**Figure 2:**
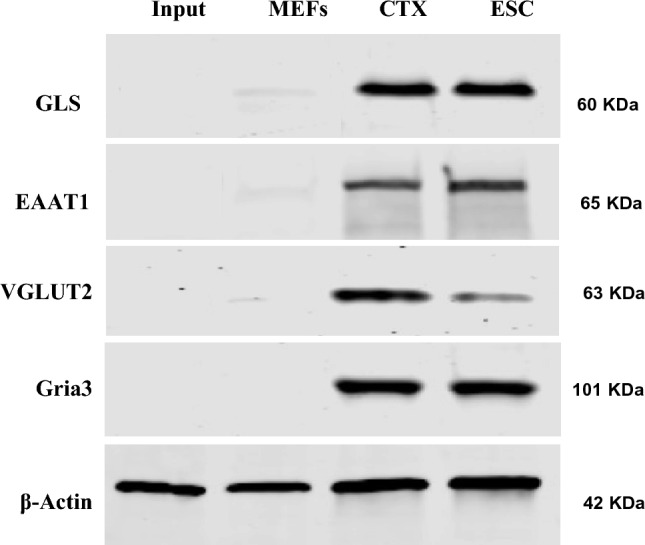
Western blot analysis for GLS, VGLUT2, EAAT1 and Gria3 in ES cells. Western blot analysis demonstrates the presence of GLS, VGLUT2, EAAT1, and Gria3 proteins in ES cells. CTX (cerebral cortex tissue) as positive control, Input as blank control, MEFs as negative control, β-actin was used in experiments as loading control. Original blots are presented in Supplementary Fig. 2.

**Figure 3:**
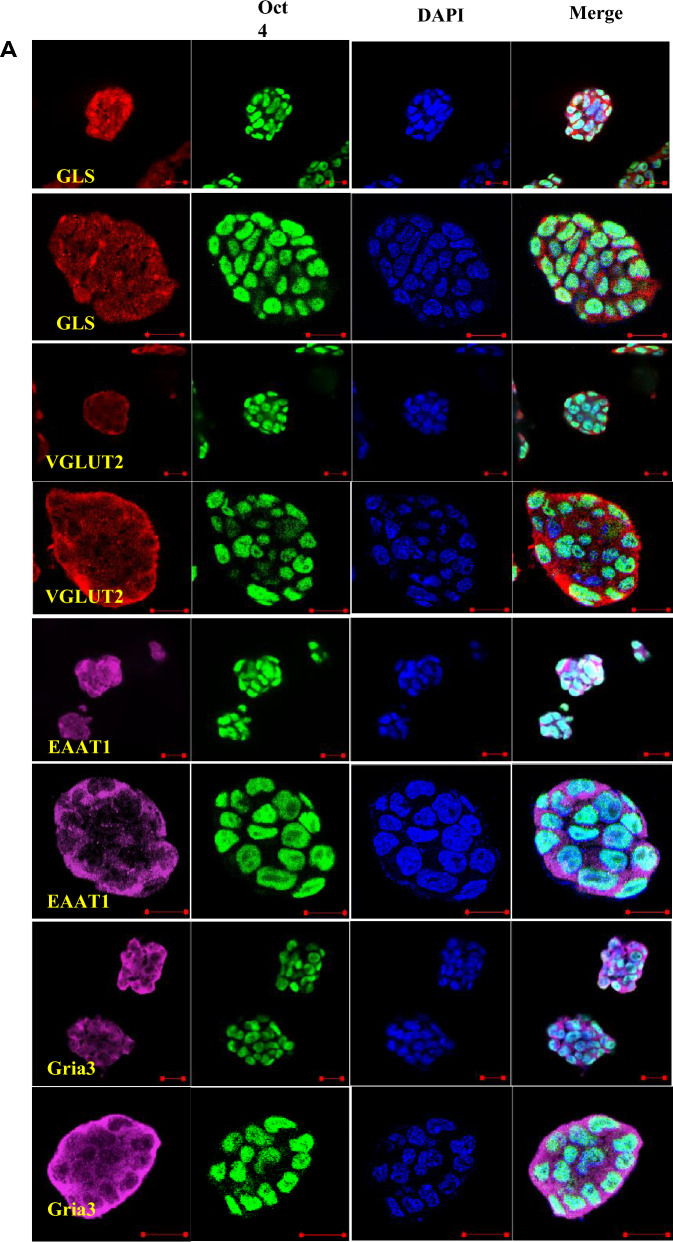

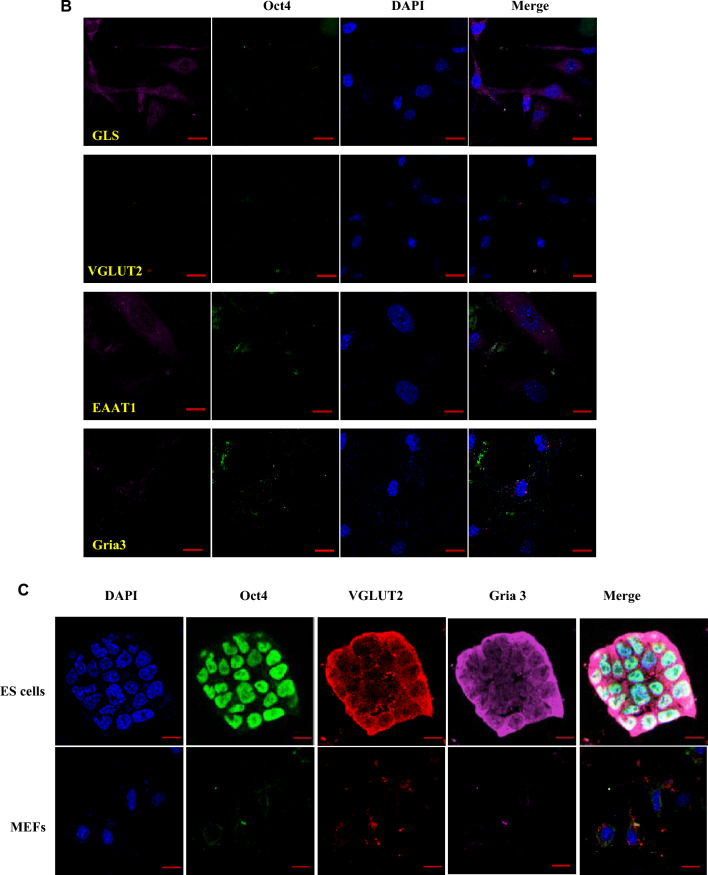
Triple immunostaining confocal images of the glutamatergic transmission output, input and reuptake components in ES cells. DAPI represents cell nucleus position, Oct4 is a pluripotent marker, GLS is the glutamate synthesis enzymes, VGLUT2 is the vesicular excitatory amino acid transporter, EAAT1 is the cell membrane transporters, Gria3 is the glutamate ionotropic receptor subtypes. MEFs are used as control. Merged images were taken at the exposure time appropriate for each wavelength. All experiments were repeated more than three times. [(**A**) ES cells, Scale bar, Multiple ES cells clone 10 μm; single ES cell clone 20 μm. (**B**) MEFs, Scale bar, 10 μm. (**C**) Colocalization of glutamatergic transmission output and input components in ES cells and MEFs, Scale bar, 10 μm].

RT-PCR analysis was performed to detect the transcription of glutamatergic signaling components in ES cells. As shown in Fig. 1, the glutamatergic signaling output components, including GLS, VGLUT1, VGLUT2, and VGLUT3 (Fig. 1A), signaling reuptake components, such as glutamate transporters EAAT1, EAAT4, and EAAT5 (Fig. 1A), and signaling input components, including AMPA receptor subunits Gria1/3/4, Delta receptor subunit Grid1/2, Kainate receptor subunits Grik2/3/4/5, NMDA receptor subunits Grin1/2a/2d, and mGluR subtypes Grm1/2/4/6 (Fig. 1B,C), were transcribed in ES cells. To further confirm the presence of the glutamatergic transmitter system in ES cells, GLS, VGLUT2, EAAT1, and Gria3 were selected as representatives to examine the protein level by performing Western blotting. Western blot analysis confirmed the presence of GLS, VGLUT2, EAAT1, and Gria3 proteins in ES cells (Fig. 2). The expression and cellular distribution of GLS, VGLUT2, EAAT1, and Gria3 in ES cells were further confirmed by confocal microscopy images of immunofluorescent staining and the cells were immunopositive for the pluripotent marker Oct4 (Fig. 3A). On the contrary, we did not detect the presence of other glutamatergic signaling components in MEFs. Instead, we observed a low level of glutaminase expression in these cells, and none of the cells exhibited immunopositivity for Oct4 (Fig. 3B). These results suggest that the glutamatergic transmission input, output, and reuptake/termination components are expressed in ES cells.

As ES cells have both glutamatergic transmitter output- and input-components, we hypothesized that ES cells release glutamate and may act on glutamate receptors on the same cell (autocrine) and/or neighboring cells (paracrine). To investigate this, we performed triple immunofluorescence staining analysis by confocal microscopy and found that VGLUT2 colocalized with Gria3, as well as the pluripotent marker Oct4 in undifferentiated ES cells (Fig. 3C). These components were absent in MEFs. VGLUT is responsible for the accumulation of l-glutamate in synaptic vesicles in glutamatergic neurons^31^. These results demonstrate that glutamatergic output and input components are colocalized in ES cells. Our findings suggest that VGLUT2 localizes preferentially to synaptic-like microvesicles and is excluded from glutamate in undifferentiated ES cells. By binding to AMPA/kainate, NMDA, or metabotropic glutamate receptors, glutamate can act on the same cell (autocrine) and/or neighboring cells (paracrine).

### ES cells express functional Ca^2+^-permeable glutamate receptors

**Figure 4:**
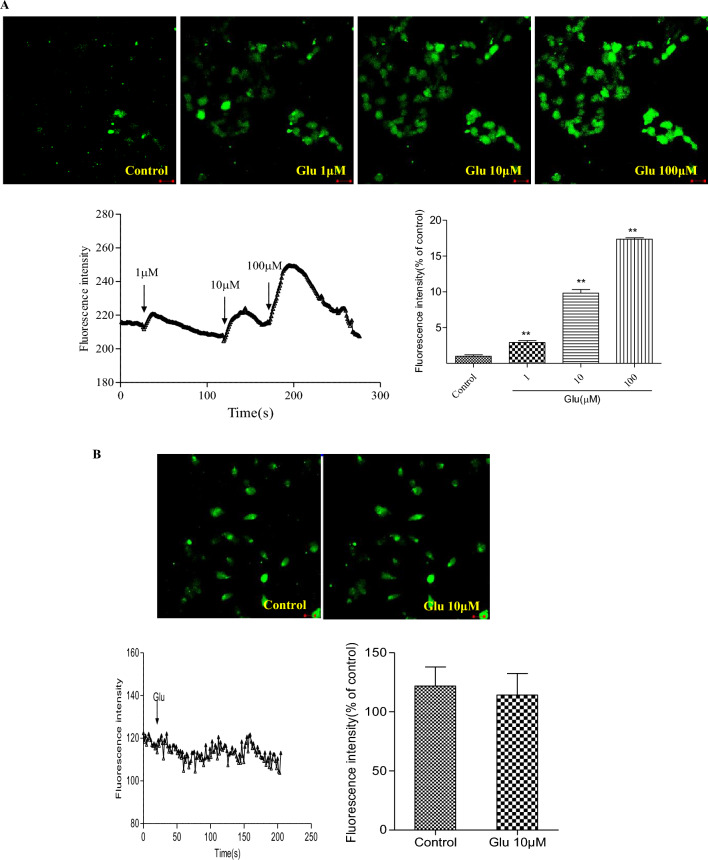
Excitation-induced Ca^2+^ influx in cultured ES cells. (**A**) Confocal image showing Ca^2+^ responses to 1 μM, 10 μM, and 100 μM glutamate in ES cells. (**B**) NIH 3T3 cells with 10μM glutamate did not induce any changes in intracellular Ca^2+^ concentration. Fluorescence intensity are expressed as mean ± SEM, n = 3 in duplicate for each cells. *Glu* glutamate. All experiments were repeated more than three times. **P < 0.01 as compared with control. Scale bar, 10 μm.

The presence of ionotropic and metabotropic glutamate receptors in ES cells does not necessarily imply their functionality. To demonstrate the functionality of glutamate receptors in ES cells, we measured changes in cytoplasmic free Ca^2+^ concentration ([Ca^2+^]i) following stimulation with glutamate or glutamate receptor antagonists, using fura-4-based digital imaging. We found that glutamate induces a sharp increase in fluorescence in ES cells, and this effect is dose-dependent (Fig. 4A). Specifically, 1 μM, 10 μM, and 100 μM glutamate led to a rapid and transient increase in intracellular Ca^2+^ concentration. In contrast, treatment of NIH 3T3 cells with 10 μM glutamate did not induce any changes in intracellular Ca^2+^ concentration (Fig. 4B).

**Figure 5:**
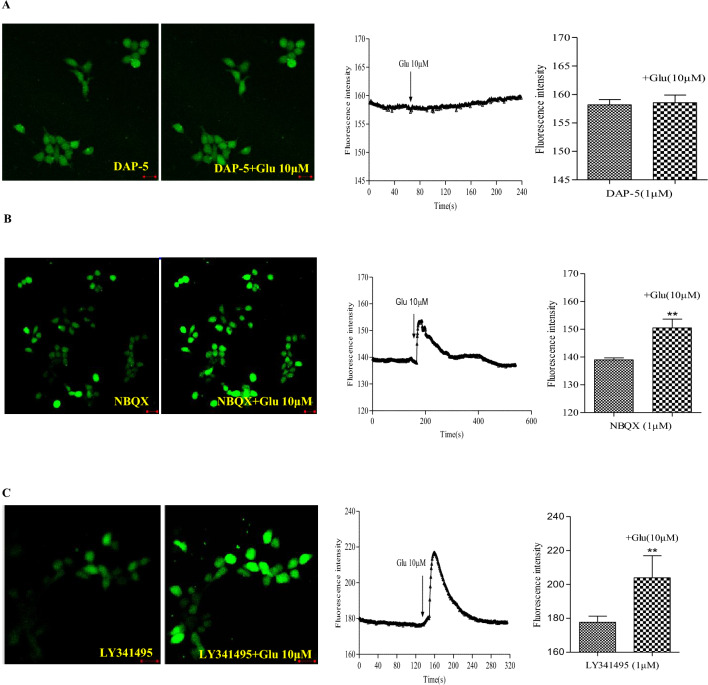
Effect of the glutamate receptor antagonists on Ca^2+^ influx in cultured ES cells. (**A**) Inbubate the cells with 1 μM DAP-5 for 15 min before 10 μM glutamate treatment. (**B**) Inbubate the cell with 1μM NBQX for 15 min after 10 μM glutamate treatment. (**C**) Incubate the cell with 1 μM LY341495 for 15 min after 10 μM glutamate treatment. Fluorescence intensity are expressed as mean ± SEM, n = 3 in duplicate for each cells. *Glu* glutamate. All experiments were repeated more than three times. Scale bar, 10 μm. **P < 0.01 as compared with absence of 10 μM glutamate.

To determine which type of glutamate receptor induces Ca^2+^ influx, we applied specific glutamate receptor antagonists NBQX (1 μM), DAP-5 (1 μM), and LY341495 (1 μM) before exposing the cells to glutamate (10 μM). Our data showed that 1 μM DAP-5 completely abolished the glutamate-induced Ca^2+^ transients (Fig. 5A), indicating the presence of functional NMDA glutamate receptors in ES cells. However, 1 μM NBQX and 1 μM LY341495 did not affect the Ca^2+^ influx (Fig. 5B,C). These experiments demonstrate that ES cells express functional Ca^2+^-permeable glutamate receptors, specifically the NMDA receptor subtype.

### ES cells release glutamate in a tonic manner

**Figure 6:**
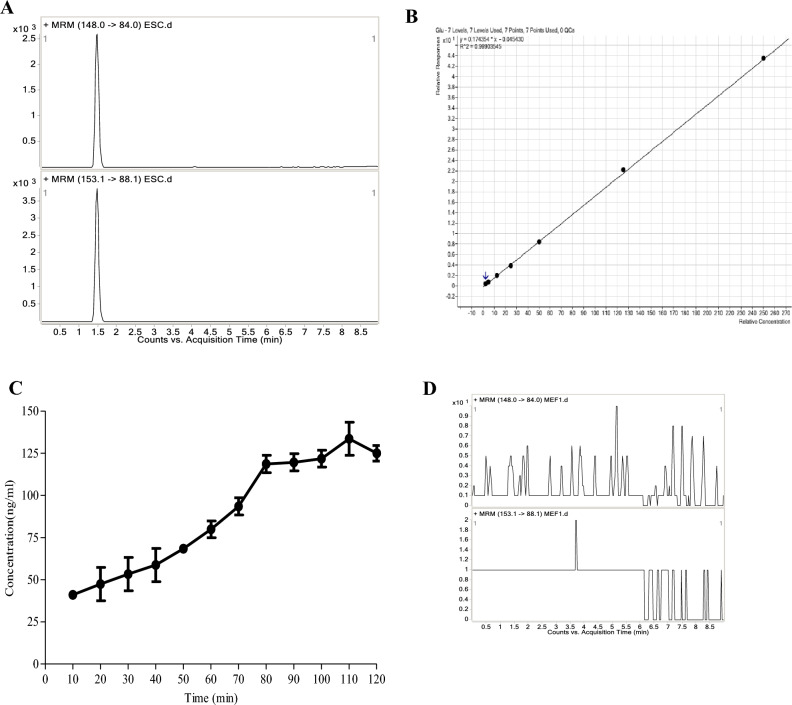
Release of glutamate by ES cells determined by MD–HPLC–MS–MS. (**A**) Representative MRM chromatograms for glutamate (up panel) and IS (down panel) spiked with glutamate at lower limit of quantitation (LLOQ) level. (**B**) Typical daily calibration curve for glutamate based on 7 standard solutions. (**C**) Time-concentration profile of glutamate released from ES cells. (**D**) Representative MRM chromatograms of the supernatants of MEFs. Concentration are expressed as mean ± SEM, n = 3 in duplicate for each cells.

Given that ES cells express components of a neuronal-like glutamatergic system, we investigated whether ES cells are capable of synthesizing and releasing glutamate. To directly demonstrate this, we used MD–HPLC–MS–MS to quantitatively monitor glutamate release from ES cells. Our results show that ES cells release glutamate in a tonic manner, with the concentration in the extracellular space increasing from 40.98 ± 3.72 ng/mL at 10 min to 79.88 ± 7.06 ng/mL at 60 min and reaching 133.57 ± 13.88 ng/mL at 110 min (Fig. 6). In contrast, no glutamate release was detected in MEFs. These data suggest that ES cells continuously release glutamate into the extracellular space until a balance is achieved between release and reuptake of the transmitter, and that glutamate release is specific to ES cells.

### Effects of endogenous glutamate signaling on ES cell proliferation and cell cycle

**Figure 7:**
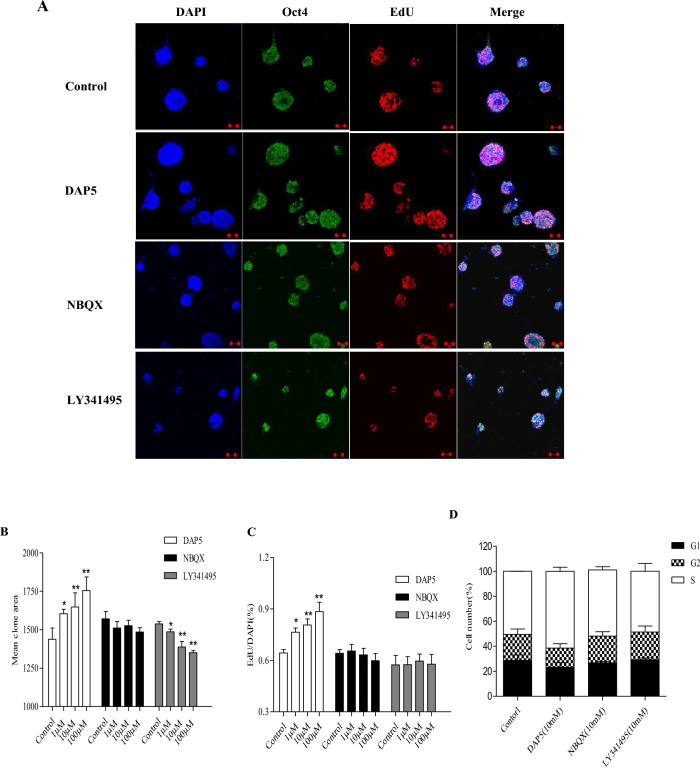
Antagonists of glutamate receptor influence ES cell proliferation. ES cells cultures were treated with different concentration of DAP5, NBQX and LY341495 for 24 h. (**A**) Confocal images of Oct4, EdU and DAPI in ES cells cultures were treated with DAP5, NBQX and LY341495 for 24 h. (**B**) The comparison of mean clone area in different groups. (**C**) Representative percentage of EdU/DAPI in different groups. (**D**) Antagonists of glutamate receptor influence ES cells cycle distribution. ES cells cultures were treated with 10 μM DAP5, NBQX and LY341495 for 24 h. Values are mean ± SEM of three independent experiments and are presented as a percentage of cells in each of the three phases of the cell cycle. *P < 0.05, **P < 0.01 as compared with control, Scale bar, 10 μm.

To investigate the precise function of the endogenous glutamate autocrine system in ES cells, we examined whether glutamate release via ionotropic and metabotropic receptors may be functionally involved in ES cells proliferation and cell cycle. High content screening analysis of 5-ethynyl-2′-deoxyuridine (EDU) incorporation and flow cytometry technology were employed to examine the effects of glutamate receptor antagonists NBQX, D-AP5, and LY341495 on ES cells, specifically regarding their G1-S-phase transition. Upon microscopic examination, treatment with DAP5 resulted in a significant increase in the mean clone area of ES cells compared to the control group. Conversely, NBQX had a minor impact on the mean cloning area of ES cells. Notably, treatment with LY341495 led to a notable decrease in the clone area (Fig. 7A,B). DAP5 also exhibited the ability to enhance the staining of EdU-incorporated positive cells, with a more pronounced effect observed at higher dosages of DAP5. On the other hand, LY341495 and NBQX did not significantly affect the incorporation of EdU in undifferentiated ES cells (Fig. 7C). Furthermore, D-AP5 demonstrated an increase in the proportion of S-phase cells in ES cells, indicating its role in promoting ES cell proliferation. In contrast, LY341495 and NBQX had no effect on the proliferation of ES cells (Fig. 7D). These findings suggest that endogenous glutamate signaling via ionotropic NMDA receptors may play a role in promoting ES cell proliferation.

## Discussion

Glutamate signaling, traditionally associated with neurotransmission, has been increasingly recognized for its roles beyond the nervous system, extending to non-neuronal cells in peripheral tissues, including those outside the brain^26,32^. Remarkably, a prototypical glutamatergic system has been identified in the islets of Langerhans, where VGLUT, EAAT, and multiple GluR expressed in pancreatic α and β cells modulate the secretion of glucagon and insulin^33–35^. Furthermore, evidence suggests that glutamatergic signaling plays a role in regulating bone catabolism, as osteoclasts express VGLUT and secrete glutamate to suppress transcytosis through mGluR, thereby modulating bone resorption and formation^36–38^. Glutamatergic components have also been identified in various other peripheral tissues such as the heart, kidney, lungs, ovary, and testis. However, our understanding of glutamatergic transmission in these tissues remains limited compared to the central nervous system^39^. Despite the significant progress in investigating glutamatergic signaling in peripheral tissues, our knowledge of its role in ES cells is relatively restricted and has primarily focused on mGluR^28–30^. In our study, we have discovered ES cells possess the complete repertoire of components required for glutamatergic transmission. These components include the enzymes responsible for glutamate synthesis, vesicular glutamate transporters, cell membrane glutamate transporters, as well as both ionotropic and metabotropic glutamate receptors. Importantly, we have detected the presence of functional receptors and observed colocalization of output-input pairs within the same cell. Furthermore, we have demonstrated that the signaling molecule itself, glutamate, can be released from the cells. Blocking the receptors with specific antagonists, which lack intrinsic activity themselves, significantly affected receptor-mediated intracellular signaling transduction and cell function. This intriguing finding suggests that ES cells have the capacity to regulate themselves by establishing a glutamatergic auto-niche. These findings strongly indicate the existence of an autocrine/paracrine mechanism in the glutamatergic system of ES cells. Specifically, ES cells can release glutamate, to which they themselves respond in an autocrine manner, or neighboring cells respond in a paracrine manner. While we observed minimal expression of glutaminase in MEFs, this low level of glutaminase expression can be attributed to several factors. MEFs primarily rely on glycolysis and oxidative phosphorylation as their primary metabolic pathways for energy production, and they may not necessitate high levels of glutaminase expression due to their distinct metabolic demands and functions compared to glutamate-producing cells. Instead, MEFs may predominantly utilize glutamine for other essential cellular processes, such as nucleotide biosynthesis. Furthermore, the gene expression profiles of MEFs, including that of glutaminase, can be influenced by their culture conditions, which may include the presence of ES cell. These specific culture conditions have the potential to modulate the expression of genes involved in glutamate metabolism^40,41^.

Recent investigations have provided additional evidence supporting the existence of glutamatergic components within ES cells, including the expression of glutamate receptors and transporters. Notably, recent studies have emphasized the significance of NMDA receptors in the process of neuronal differentiation in human ES cells^42,43^, while also demonstrating the capability of glutamate to induce neural differentiation through metabolic pathways^44,45^. Additionally, the activation of glutamate receptors has been shown to promote proliferation of human ES cells via the MAPK pathway^46^. These recent findings further support the presence of a functional glutamatergic system in ES cells and its potential role in regulating their behavior. However, it has not been demonstrated whether ES cells can produce and release glutamate. Our study provides the first direct evidence that ES cells are capable of releasing glutamate in a tonic manner, as shown by the increase in glutamate concentration over time. Moreover, this glutamate release was specific to ES cells, as no glutamate was detected in MEFs.

The co-localization of glutamatergic output- and input-components in undifferentiated ES cells is a novel finding. Triple immunofluorescence staining analysis revealed the co-localization of pluripotent marker Oct4, glutamatergic marker VGLUT2^47,48^, and glutamate receptor subunit Gria3 in ES cells. VGLUT2 is responsible for the accumulation of l-glutamate in synaptic vesicles in glutamatergic neurons, and Gria3 encodes a subunit of the AMPA receptor. The co-localization of these two components suggests that glutamate may be released from synaptic-like microvesicles in ES cells and may then act on AMPA receptors on the same cell or neighboring cells. Oct4 is a pluripotent marker expressed in undifferentiated ES cells and its co-localization with VGLUT2 and Gria3 suggests that the glutamatergic system may play a role in maintaining pluripotency in ES cells. These confirming the possibility that the transmitter binds its cognate receptor in a cell that produce the transmitter and endogenously produced transmitter functions/effects in an autocrine or paracrine manner.

The mechanism by which NMDA receptor activation promotes ES cell proliferation remains to be elucidated. In our study, one possibility is that NMDA receptor activation leads to an increase in intracellular calcium, which in turn activates calcium-dependent signaling pathways that promote cell proliferation, this is consistent with previous literature reports^49^. Another possibility is that NMDA receptor activation may alter the expression of genes that are involved in cell proliferation. NMDA receptor activation can activate the MAPK/ERK signaling pathway, which is known to promote cell proliferation in various cell types^50^. These studies provide some insights into the potential mechanisms underlying NMDA receptor-mediated ES cell proliferation, but further research is still needed to fully understand the complex signaling pathways involved. The lack of an effect of LY341495 and NBQX on ES cell proliferation is surprising, given that previous studies have shown that glutamate can promote cell proliferation through activation of AMPA and metabotropic glutamate receptors^51,52^. One possible explanation for the lack of effect of LY341495 and NBQX on ES cell proliferation in our study could be due to the specific characteristics of ES cells. ES cells have a unique set of receptors and signaling pathways that may differ from those present in mature neurons or other cell types. For example, ES cells express high levels of the NMDA receptor subunit NR2B, which has been shown to be involved in the regulation of ES cell self-renewal and pluripotency. It is possible that the effects of glutamate signaling on ES cell proliferation are mediated through NR2B, rather than AMPA or metabotropic glutamate receptors. Another possible explanation for the lack of effect of LY341495 and NBQX on ES cell proliferation is that the autocrine glutamate signaling system in ES cells may serve a different function than promoting cell proliferation. It is well established that glutamate is a key neurotransmitter in the brain and plays important roles in synaptic plasticity, learning and memory, and other physiological processes. It is possible that the glutamate autocrine system in ES cells has a similar function in regulating self-renewal and differentiation, rather than promoting cell proliferation.

Stem cells are regulated by a combination of intrinsic and extrinsic factors, including transcription factors, epigenetic control, microRNA regulators, growth factors, and cytokines^53–55^. Autocrine and paracrine signals, such as FGFs, Activin, Wnt, and LIF, play crucial roles in stem cell survival, self-renewal, growth, differentiation, and embryonic development^56–58^. Interestingly, ES cells have the ability to establish a glutamatergic auto-niche, whereby they release and respond to small signaling molecules, regulating both themselves and neighboring cells. Similar effects have been observed with γ-Aminobutyric acid (GABA) in stem^59,60^ and cancer cell niches^61–65^. Previous research has revealed the presence of functional glutamatergic components in various multipotent and pluripotent cell types, shedding light on the diverse roles of glutamate signaling beyond traditional neurotransmission. While our study presents evidence of a glutamate autocrine signaling system in the R1 ES cell line, it is essential to acknowledge the broader landscape of glutamate signaling in similar cell contexts. In neural stem cells (NSCs), for instance, glutamatergic transmission has been extensively studied. Several studies have demonstrated the expression of ionotropic glutamate receptors, such as NMDA receptors, in NSCs, and their involvement in modulating cell proliferation and differentiation during neurogenesis^66,67^. Additionally, pluripotent cells, including induced pluripotent stem cells (iPSCs), have been investigated in the context of glutamate signaling. Researchers have reported the presence of both ionotropic and metabotropic glutamate receptors in iPSCs, with implications for their self-renewal and differentiation capacities. These findings suggest that glutamate may play a pivotal role in regulating pluripotent cell behavior beyond its role as a neurotransmitter^68,69^. Regarding the mechanisms of glutamate release from non-neuronal cells, studies have suggested vesicular release, similar to neurons, as a possible mode of glutamate secretion. However, the precise mechanisms underlying vesicular release in non-neuronal cells and its role as a trophic factor require further investigation. It is evident that glutamate's actions extend beyond classical neurotransmission, influencing cell proliferation, differentiation, and function in various cell types^26,70^.

In conclusion, our study provides the first evidence of an endogenous glutamate autocrine signaling system in ES cells, suggesting its involvement in regulating ES cell proliferation. These glutamatergic systems, present in the earliest stage pluripotent cells, control fundamental cellular functions beyond synaptic transmission in the “pre-nervous” stage. The identification of glutamatergic systems in ES cells has several implications: (1) Glutamate acts as an external factor modulating stem cell behavior. (2) ES cells, through autocrine and paracrine mechanisms, create and regulate a niche involving themselves and neighboring cells, fine-tuning via autoreceptors and signal termination mechanisms. (3) The components of these systems could serve as potential targets for optimizing cell replacement therapies^71^. (4) Neuroactive drugs commonly used in clinical practice, targeting the components of glutamatergic systems, have the potential to impact developing cells and embryos, potentially leading to teratogenesis and birth defects^72–74^.

### Supplementary Information

Supplementary Figures.

## Data Availability

All data generated or analysed during this study are included in this published article.
