# Supplementary material for: Glutamate secretion by embryonic stem cells as an autocrine signal to promote proliferation
Source: Sci Rep. 2023 Nov 4;13:19069. doi: 10.1038/s41598-023-46477-2 (PMC10625544; doi:10.1038/s41598-023-46477-2)
Supplement: Supplementary file 1 — Supplementary Figures. [file 41598_2023_46477_MOESM1_ESM.ppt]

## Slide 1
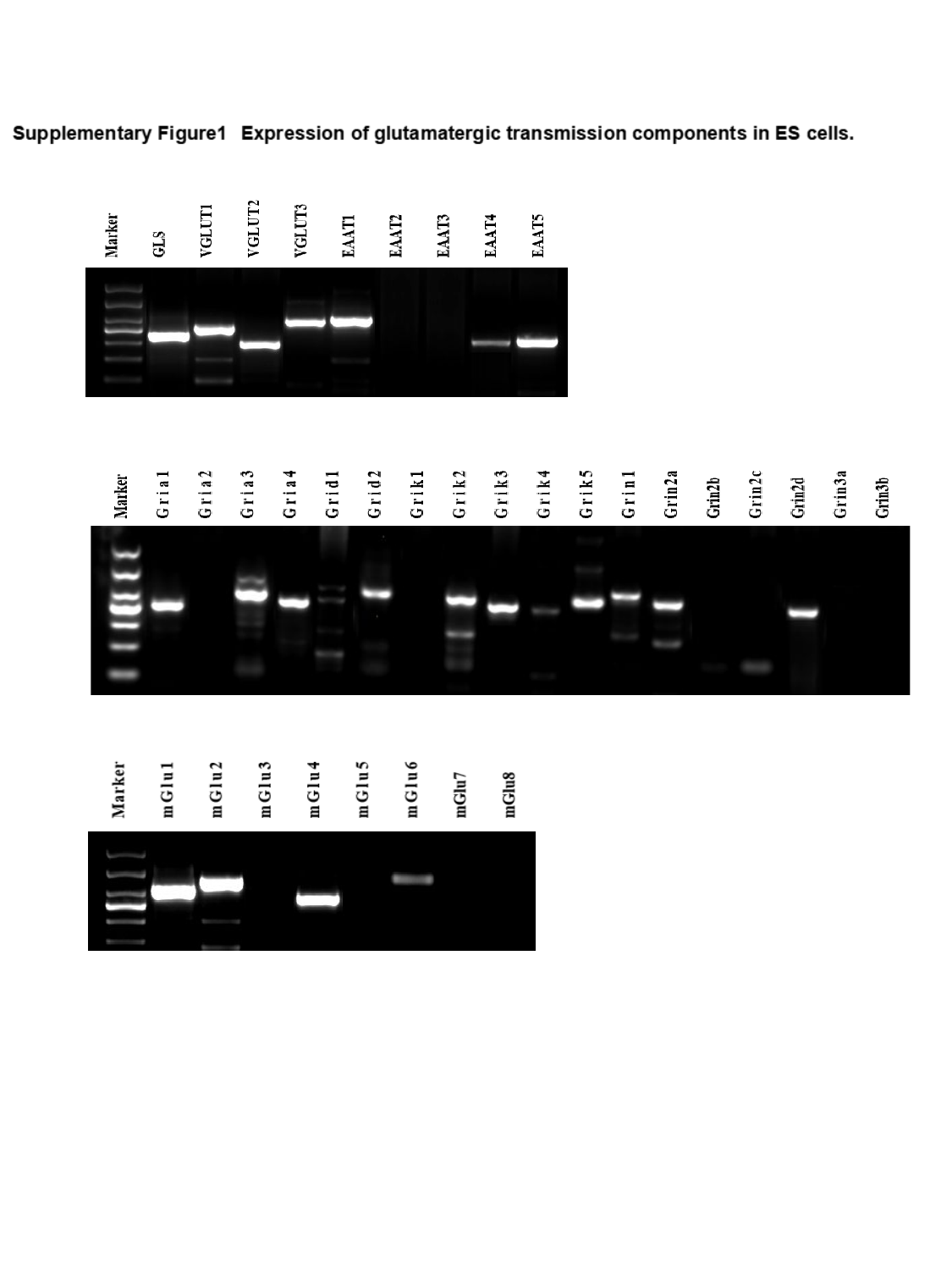

## Slide 2
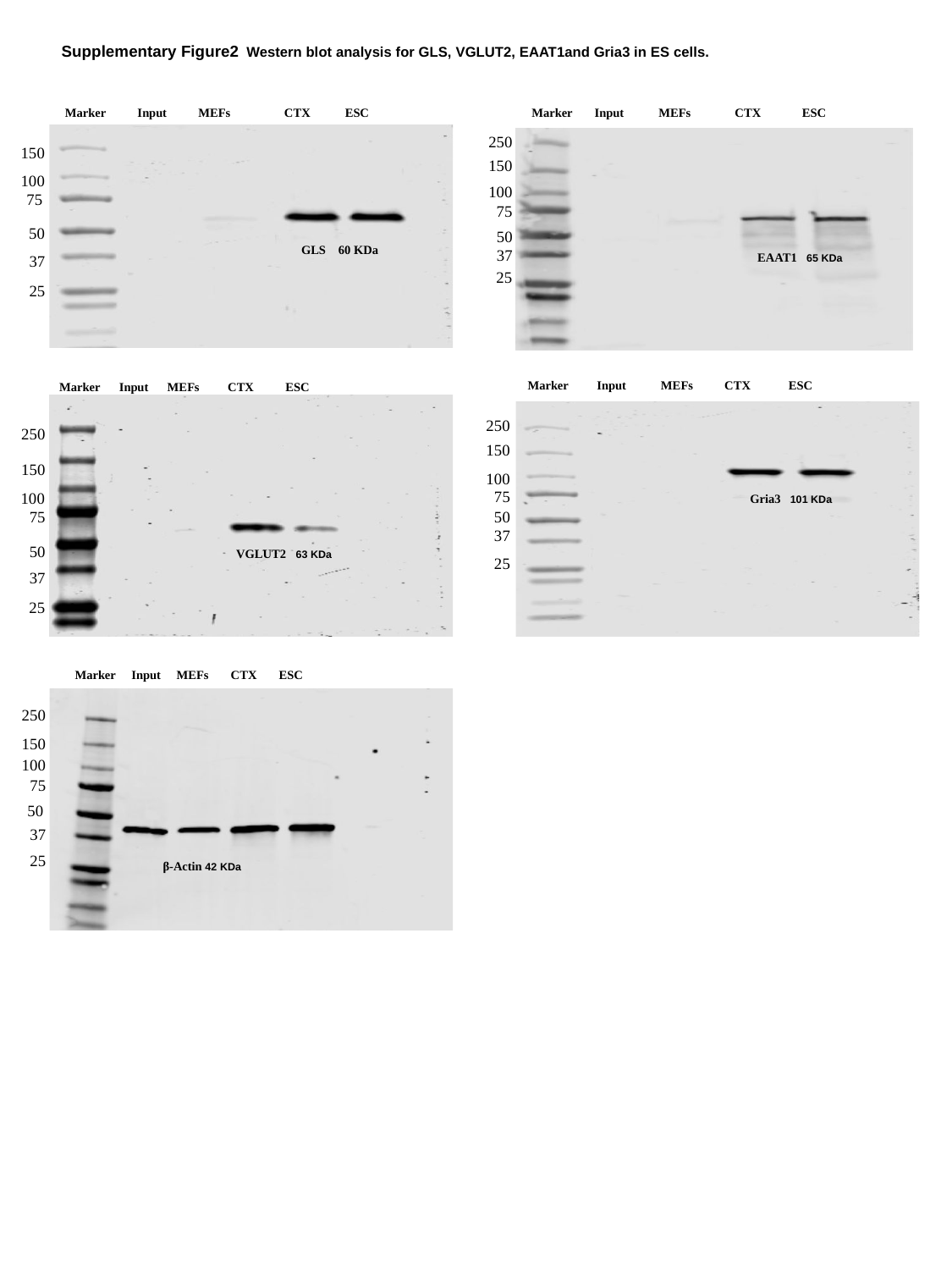

Supplementary Figure2 Western blot analysis for GLS, VGLUT2, EAAT1and Gria3 in ES cells.
 Marker Input MEFs CTX ESC
 Marker Input MEFs CTX ESC
250
150
100
 75
 50
 37
 25
150
100
 75
 50
 37
 25
 GLS 60 KDa
 EAAT1 65 KDa
 Marker Input MEFs CTX ESC
250
150
100
 75
 50
 37
 25
 Gria3 101 KDa
 Marker Input MEFs CTX ESC
250
150
100
 75
 50
 37
 25
VGLUT2 63 KDa
 Marker Input MEFs CTX ESC
250
150
100
 75
 50
 37
 25
 β-Actin 42 KDa
